# Quercetins, Chlorogenic Acids and Their Colon Metabolites Inhibit Colon Cancer Cell Proliferation at Physiologically Relevant Concentrations

**DOI:** 10.3390/ijms241512265

**Published:** 2023-07-31

**Authors:** Alice Cattivelli, Angela Conte, Davide Tagliazucchi

**Affiliations:** Department of Life Sciences, University of Modena and Reggio Emilia, Via Amendola, 2-Pad. Besta, 42100 Reggio Emilia, Italy; alice.cattivelli@unimore.it (A.C.); angela.conte@unimore.it (A.C.)

**Keywords:** phenolic compounds, mass spectrometry, colon cancer, anti-proliferative activity, gut microbiota, metabolism

## Abstract

Several studies have suggested that a phenolic-rich diet may be protective against colon cancer. Most phenolic compounds are not absorbed in the small intestine and reach the colon where they are metabolized by gut microbiota in simple phenolic acids. In this study, the anti-proliferative activity of quercetins, chlorogenic acids, their colon metabolites and mixtures of parent compounds/metabolites was assessed by using two colon cancer cell lines (Caco-2 and SW480) at physiologically relevant concentrations. Chlorogenic acids, quercetin and the metabolite 3-(3′,4′-dihydroxyphenyl)acetic acid exerted remarkable anti-proliferative activity against Caco-2, whereas quercetin derivatives and metabolites were the most active against SW480. Tested compounds arrested the cell cycle at the S phase in both the cell lines. The mixtures of parent compounds/metabolites, which mimic the colon human metabotypes that slowly or rapidly metabolize the parent compounds, similarly inhibited cell growth. SW480 cells metabolized parent phenolic compounds more rapidly and extensively than Caco-2, whereas colon metabolites were more stable. These results suggest that dietary phenolic compounds exert an anti-proliferative effect against human colon cancer cells that can be further sustained by the colon metabolites. Therefore, gut microbiota metabolism of phenolic compounds may be of paramount importance in explaining the protective effect of phenolic-rich foods against colon cancer.

## 1. Introduction

Colorectal cancer is one of the most frequent forms of cancer, playing an important role in mortality, lifestyle and economic costs worldwide [1]. Colorectal cancer is primarily affected by lifestyle (such as diets and low physical activity), genetic predisposition, and the presence of chronic intestinal inflammation conditions (such as inflammatory bowel diseases). This pathology is characterized by a low heritability level, with only 12–35% of CRC cases attributable to genetic predisposition, reflecting the crucial role of dietary and environmental factors [2]. Colorectal cancer prevention is one of the most essential priorities in public health, especially considering that modifiable lifestyle factors had a pivotal role in the occurrence and progression of colorectal cancer [3]. Several epidemiological and observational studies have highlighted the strong impact of diet on the risk of colorectal cancer [4,5,6,7]. Dietary factors and the type of diet are among the main determinants in the development of colorectal cancer [4]. For example, diets rich in fibers, calcium and dairy products as well as a plant-based diet rich in phenolic compounds can decrease the risk of developing colorectal cancer [4,5,8,9]. Vice versa, a low-fiber diet associated with a high intake of red and processed meat may increase the risk of the onset of colorectal cancer [4,5,8,9].

Among the different dietary patterns, the Mediterranean diet is widely recognized as a healthy diet that may be protective against the onset of several chronic diseases, including cancer [10,11]. For example, populations with a high adherence to the Mediterranean diet displayed a lower incidence of colorectal cancer, suggesting that the Mediterranean diet may be chemopreventive against this type of cancer [10,12,13]. Indeed, the Mediterranean diet is characterized by a high intake of vegetables, fiber-rich foods, fresh fruits and legumes together with a low intake of red and processed meat [11].

The beneficial consequences of a Mediterranean diet—and, more generally, a diet rich in vegetables and fruits—in preventing the onset of colorectal cancer may be in part ascribable to the high content in phenolic compounds of these foods [8,10]. For example, Zamora-Ros et al. [14] found a positive association between the intake of phenolic acids (mainly hydroxycinnamic acids) and a reduction in colon cancer, whereas Chang et al. [15] suggested that a high intake of flavonoids (especially flavonols) may decrease the onset of colon cancer.

Hydroxycinnamic acids are an important class of phenolic compounds which are mainly found in nature esterified with one or more quinic acid moieties, producing the so-called chlorogenic acids [16]. The most important chlorogenic acids found in nature are caffeoylquinic, feruloylquinic and dicaffeoylquinic acids [17]. In the context of the Mediterranean diet, they may account for 25–35% of total phenolic compound intake [18,19]. The main dietary sources of chlorogenic acids in the Mediterranean diet are coffee, vegetables (such as eggplant and carrots) and fruits (such as cherries and apples) [19,20,21].

Flavonols are another widespread class of dietary phenolic compounds that are mainly found in nature in the form of glycoside derivatives [22]. Flavonols may account for 7–15% of total phenolic compounds in the Mediterranean diet, where the main sources are fruits and vegetables and, in particular, onions, apples, berries and red wine [18,23]. The principal flavonols identified in vegetables are glycosylated quercetin derivatives, such as quercetin-3-*O*-glucoside and quercetin-3-*O*-rutinoside, as well as quercetin-4′-*O*-glucoside and quercetin-3-*O*-glucoside-4′-*O*-glucoside in onion [22,23].

Several meta-analyses of epidemiological studies as well as animal studies provide support for the protective effect of flavonols and hydroxycinnamic acids against colorectal cancer [15,24,25,26]. Nevertheless, the data extracted from human intervention studies are still inconclusive owing to the considerable inter-individual variability due largely to the different gut microbiota metabolism of phenolic compounds among individuals.

Quercetin and quercetin derivatives reaching the colon are subjected to microbiota metabolism that involves a deglycosylation reaction, followed by C-ring fission and β-oxidation generating some low-molecular-weight phenolic acids, mainly 3-(3′-hydroxyphenyl)propanoic acid, 3-(3′-hydroxyphenyl)acetic acid and 3-(3′,4′-dihydroxyphenyl)acetic acid [27,28]. Similarly, the colon metabolism of chlorogenic acids starts with the removal of the quinic acid moiety followed by reduction and de-hydroxylation, which results in the accumulation of 3-(3′-hydroxyphenyl)propanoic acid [29,30]. However, the amount and the type of the produced metabolites greatly differed among individuals depending on the microbiota composition [17,31]. Therefore, both the parent compounds and the microbial metabolites co-exist in the colon, and the ratio of parent compounds/metabolites strongly depends on the individual microbiota composition and on the ability to produce high or low amounts of phenolic metabolites and, therefore, on inter-individual variability and metabotype [32,33]. In the low-producers metabotype (i.e., individuals that produce low amounts of colon metabolites) the ratio of parent compounds/metabolites is strongly shifted towards the parent compounds, whereas in the high-producers metabotype (i.e., individuals that produce high amounts of colon metabolites) the equilibrium is directed towards a higher amount of colon metabolites.

Thus, the aim of this work was to study the anti-proliferative activity and the cell metabolism of selected quercetin derivatives (quercetin, quercetin-4′-*O*-glucoside and quercetin-3-*O*-glucoside-4′-*O*-glucoside) and chlorogenic acids (3-*O*-caffeoylquinic acid, 5-*O*-caffeoylquinic acid and 3,5-di-*O*-caffeoylquinic acid) as well as their most important colon metabolites (3-(3′-hydroxyphenyl)propanoic acid, 3-(3′-hydroxyphenyl)acetic acid and 3-(3′,4′-dihydroxyphenyl)acetic acid) using colon adenocarcinoma Caco-2 and SW480 cell lines. Indeed, to understand the possible role of inter-individual variability, six different mixes were prepared, mimicking the low-producers (high amounts of parent compounds and low amounts of metabolites) and the high-producers (low amounts of parent compounds and high amounts of metabolites) metabotypes in comparison with a third mix with equimolar amounts of parent compounds and metabolites. The aim was to understand if colon metabolites may retain anti-proliferative activity against colon cancer cells sustaining the effect of the parent compounds and to shed light on the possible different impact of the diverse human metabotypes on the anti-cancer potential of phenolic-rich vegetables.

## 2. Results

### 2.1. Anti-Proliferative Activity of Phenolic Compounds and Metabolites

**Figure 1:**
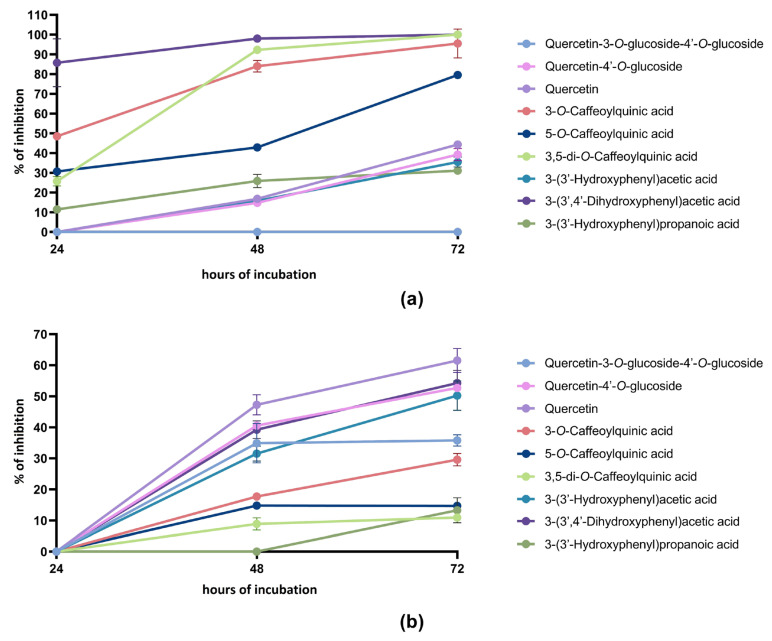
Antiproliferative activity of parent phenolic compounds and colon metabolites. All the compounds were tested at a final concentration of 100 μmol/L. (**a**) Percentage of inhibition of Caco-2 growth after 24, 48 and 72 h. (**b**) Percentage of inhibition of SW480 growth after 24, 48 and 72 h. Tested compounds were quercetins (quercetin-3-*O*-glucoside-4′-*O*-glucoside, quercetin-4′-*O*-lucoside and quercetin) and quercetin-derived colon metabolites (3-(3′-hydroxyphenyl)acetic acid, 3-(3′,4′-dihydroxyphenyl)acetic acid and 3-(3′-hydroxyphenyl)propanoic acid) as well as chlorogenic acids (3,5-di-*O*-caffeoylquinic acid, 3-*O*-caffeoylquinic acid and 5-*O*-caffeoylquinic acid) and the chlorogenic-acid-derived colon metabolite 3-(3′-hydroxyphenyl)propanoic acid. Data are presented as mean ± standard deviation.

The tested parent phenolic compounds and metabolites at 100 μmol/L concentration exerted a time-dependent anti-proliferative activity against both the colon cancer cell lines, with the only exception being quercetin-3-*O*-glucoside-4′-*O*-glucoside which did not show any activity against Caco-2 cell proliferation at any time (Figure 1a,b).

In general, quercetin derivatives were more active against SW480 cell proliferation with respect to Caco-2 proliferation. The anti-proliferative activity started to be detectable after 48 h of incubation and increased in both the cell lines after 72 h of incubation. Among quercetin derivatives, the highest activity was found in both cell lines for quercetin, followed by quercetin-4′-*O*-glucoside. For both the compounds, the anti-proliferative effect was significantly higher (*p* < 0.05) against SW480 with respect to Caco-2 both after 48 and 72 h of incubation. As already mentioned, quercetin-3-*O*-glucoside-4′-*O*-glucoside displayed anti-proliferative activity only against the SW480 cell line, although it was significantly lower (*p* < 0.05) with respect to quercetin and quercetin-4′-*O*-glucoside.

An opposite trend was observed for chlorogenic acids, since they displayed a significantly higher (*p* < 0.05) anti-proliferative activity against Caco-2 with respect to the SW480 cell line at any time. In Caco-2 cells, 3,5-di-*O*-caffeoylquinic acid, 3-*O*-caffeoylquinic acid and 5-*O*-caffeoylquinic acid were active already after 24 h of incubation, and then the anti-proliferative activity increased over time, reaching an almost complete inhibition after 72 h of incubation. The anti-proliferative activity of 5-*O*-caffeoylquinic acid was significantly lower (*p* < 0.05) than that of its positional isomer 3-*O*-caffeoylquinic acid at any time. In the case of SW480, the maximum effect was observed after 72 h of incubation for both the compounds, with 3-*O*-caffeoylquinic acid more active than 3,5-di-*O*-caffeoylquinic acid and 5-*O*-caffeoylquinic acid (29.6%, 10.9% and 14.7% of inhibition at 72 h, respectively).

A similar trend was observed also for the phenolic metabolites, which exerted a higher anti-proliferative effect (*p* < 0.05) against Caco-2 with respect to SW480 at any incubation time, except for 3-(3′-hydroxyphenyl)acetic acid. The colon metabolite 3-(3′,4′-dihydroxyphenyl)acetic acid was the tested compound that exerted the highest anti-proliferative effect against the Caco-2 cell line. The metabolite 3-(3′-hydroxyphenyl)propanoic acid was more effective than 3-(3′-hydroxyphenyl)acetic acid after 24 and 48 h of incubation with the Caco-2 cell line. However, no significant differences (*p* > 0.05) were found in the anti-proliferative activity of these two colon metabolites after 72 h of incubation with Caco-2 cells. No anti-proliferative activity was observed in the SW480 cell line for all the tested colon metabolites after 24 h of incubation. After 48 and 72 h of incubation with SW480 cells, no significant differences (*p* > 0.05) were found between the anti-proliferative activity of 3-(3′,4′-dihydroxyphenyl)acetic acid and 3-(3′-hydroxyphenyl)acetic acid. Among the tested metabolites, 3-(3′-hydroxyphenyl)propanoic acid displayed the lowest anti-proliferative activity against SW480 cells, with a recorded 13.3% of inhibition after 72 h of incubation.

The IC_50_ values were calculated for the most active compounds and data are reported in Table 1.

In the case of Caco-2 cells, the lowest IC_50_ values were calculated for the colon metabolite 3-(3′,4′-dihydroxyphenyl)acetic acid at any time of incubation. The IC_50_ values decreased according to the incubation time by 5.4 times from 24 to 48 h and by 4.9 times from 48 to 72 h. The IC_50_ values were also calculated for the three tested chlorogenic acids. At 24 h, 3-*O*-caffeoylquinic acid was more effective than 3,5-di-*O*-caffeoylquinic acid, whereas this last compound passed 3-*O*-caffeoylquinic acid in its anti-proliferative effect after 48 and 72 h of incubation. The IC_50_ value of 3-*O*-caffeoylquinic acid decreased by 2.3 times passing from 24 to 48 h of incubation, whereas no significant differences (*p* > 0.05) were detected in the IC_50_ values between 48 and 72 h of incubation.

The compound 5-*O*-caffeoylquinic acid displayed higher IC_50_ values (and therefore lower activity) than 3-*O*-caffeoylquinic acid at 24 and 48 h; however, at 72 h, the calculated IC_50_ value for 5-*O*-caffeoylquinic acid was significantly lower (*p* < 0.05) than that of 3-*O*-caffeoylquinic acid. The decrease in the IC_50_ values for 5-*O*-caffeoylquinic acid was 1.7-times from 24 to 48 h and 3.2-times from 48 to 72 h. For 3,5-di-*O*-caffeoylquinic acid, a 3.1-fold decrease in the IC_50_ value was recorded from 48 to 72 h of incubation.

In the case of SW480, the IC_50_ values were calculated only for two quercetin derivatives and two metabolites. In the case of quercetin derivatives, the lowest IC_50_ values were found for quercetin both after 48 and 72 h of incubation, with a recorded decrease of 1.5-fold passing from 48 to 72 h. A similar decrease in the IC_50_ values was observed for quercetin-4′-*O*-glucoside (1.4-fold) going from 48 to 72 h of incubation. The IC_50_ values calculated for the metabolites 3-(3′,4′-dihydroxyphenyl)acetic acid and 3-(3′-hydroxyphenyl)acetic acid after 72 h of incubation were not significantly different (*p* > 0.05).

### 2.2. Anti-Proliferative Activity of Quercetin Derivatives/Metabolites and Chlorogenic Acid/Metabolite Mixes

**Figure 2:**
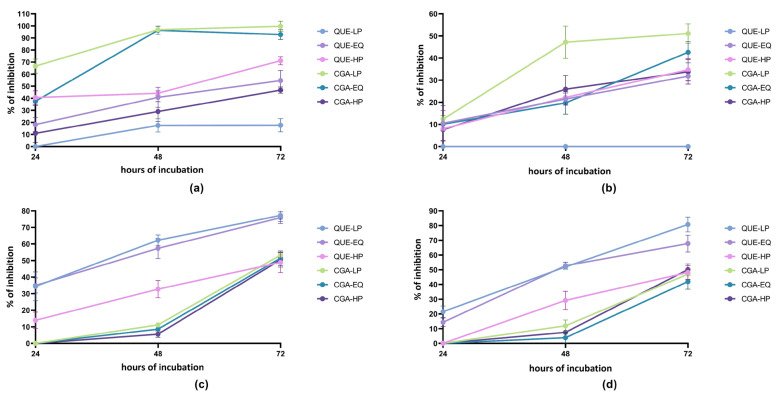
Antiproliferative activity of parent phenolic compound/metabolite mixtures. (**a**) Percentage of inhibition of Caco-2 growth after 24, 48 and 72 h by the different mixtures at a concentration of 200 μmol/L. (**b**) Percentage of inhibition of Caco-2 growth after 24, 48 and 72 h by the different mixtures at a concentration of 100 μmol/L. (**c**) Percentage of inhibition of SW480 growth after 24, 48 and 72 h by the different mixtures at a concentration of 200 μmol/L. (**d**) Percentage of inhibition of SW480 growth after 24, 48 and 72 h by the different mixtures at a concentration of 100 μmol/L. Quercetin derivative/metabolite mixtures (QUE mixes) were prepared by mixing the parent compounds quercetin-3-*O*-glucoside-4′-*O*-glucoside, quercetin-4′-*O*-glucoside and quercetin as well as the colon metabolites 3-(3′-hydroxyphenyl)acetic acid, 3-(3′,4′-dihydroxyphenyl)acetic acid and 3-(3′-hydroxyphenyl)propanoic acid. Chlorogenic acid/metabolite mixtures (CGA mixes) were prepared by mixing the parent compounds 3,5-di-*O*-caffeoylquinic acid, 3-*O*-caffeoylquinic acid and 5-*O*-caffeoylquinic acid as well as the colon metabolite 3-(3′-hydroxyphenyl)propanoic acid. The mixtures which were formulated to mimic the metabotype of low-producers of colon metabolites (QUE-LP and CGA-LP), contained 90% of parent compounds and 10% of metabolites, and those formulated to mimic the metabotype of high-producers of colon metabolites (QUE-HP and CGA-HP) contained 10% of parent compounds and 90% of metabolites. Mixes QUE-EQ and CGA-EQ contained an equimolar amount of each compound. Data are presented as mean ± standard deviation.

Exposure of Caco-2 and SW480 cell lines to the different mixes resulted in an anti-proliferative effect, the intensity of which was dependent on the cell line, mix composition, mix concentration and time of exposure (Figure 2).

As regards the quercetin derivative/metabolite mixes, the trend in the anti-proliferative activity was opposite considering the two cell lines. In the Caco-2 cell line, the cell-growth-inhibitory effect increased passing from the mix containing a higher amount of parent compounds (QUE-LP mix) to the mixture containing a higher amount of metabolites (QUE-HP mix).

This was particularly evident when the mixes were tested at a 200 μmol/L concentration. In particular, the mix QUE-HP reached 71% of cell growth inhibition after 72 h of incubation at 200 μmol/L. The opposite behavior was recorded with respect to the SW480 cell line, where the anti-proliferative activity decreased as the concentration of the metabolites in the mix increased (from QUE-LP to QUE-HP). The mixes QUE-LP and QUE-EQ attained an anti-proliferative activity of 77% and 76% after 72 h of incubation at 200 μmol/L, respectively. All this considered, the mix QUE-HP was still able to inhibit SW480 proliferation by about 50% after 72 h of incubation at 200 μmol/L.

Anti-proliferative activity was also observed for the chlorogenic acid/metabolite mixtures in both the cell lines. In the Caco-2 cell line, the highest anti-proliferative effect was observed with CGA-LP and CGA-EQ, which contained high amounts of the parent compounds. The effect was already observed after 24 h of incubation and reached values of more than 90% after 48 h of incubation. The mix with the highest amount of metabolites (CGA-HP) displayed lower inhibitory activity at any incubation time, with a maximum inhibitory activity of 47% after 72 h of incubation at 200 μmol/L.

Chlorogenic acid/metabolite mixtures did not show anti-proliferative activity after 24 h of incubation with SW480 cells, whereas they achieved the maximum effect after 72 h of incubation. No significant differences were observed among the inhibitory activities of the three mixes after 72 h of incubation with SW480 cells.

### 2.3. Cell Cycle Analysis

To obtain more information on the possible mechanism of anti-proliferative activity of phenolic compounds and metabolites, cell cycle analysis by flow cytometry was carried out on both the cell lines. Only those compounds for which an IC_50_ value was calculated were analyzed for their effect on cell cycle distribution. The active compounds were incubated for 72 h at a concentration equal to the calculated IC_50_ value.

**Figure 3:**
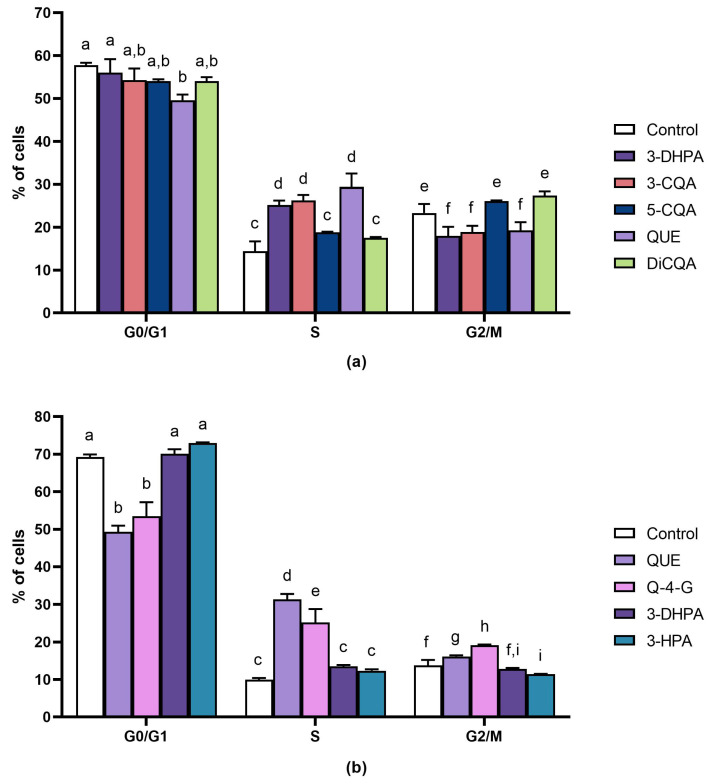
Effect of selected compounds on cell cycle progression in Caco-2 cells (**a**) and SW480 cells (**b**) after 72 h of incubation. The phases of the cell cycle are illustrated as control (cells incubated in absence of phenolic compounds) and as cells treated with the active compounds at a concentration equal to the calculated IC_50_ value. The data are expressed as mean ± SD. Significant differences were analyzed by the one-way ANOVA test. Different letters among samples denote significant differences (*p* < 0.05). 3-CQA: 3-*O*-caffeoylquinic acid; 5-CQA: 5-*O*-caffeoylquinic acid; DiCQA: 3,5-di-*O*-caffeoylquinic acid; QUE: quercetin; Q-4-G: quercetin-4′-*O*-glucoside; 3-DHPA: 3-(3′,4′-dihydroxyphenyl)acetic acid; 3-HPA: 3-(3′-hydroxyphenyl)acetic acid.

Data displayed in Figure 3a shows that, after 72 h of incubation, the percentage of control Caco-2 cells in the G0/G1, S and G2/M phases was 57.8 ± 0.6%, 14.4 ± 2.3% and 23.3 ± 2.1%, respectively. All the tested compounds clearly triggered an increase in the percentage of Caco-2 cells in the S phase. This was particularly evident after treatments with 3-*O*-caffeoylquinic acid, quercetin and the metabolite 3-(3′,4′-dihydroxyphenyl)acetic acid where the increase in the percentage of Caco-2 cells in the S phase was associated to a significant (*p* < 0.05) decrease in the percentage of Caco-2 cells in the G2/M phase. Quercetin also significantly decreased (*p* < 0.05) the percentage of Caco-2 cells in the G0/G1 phase. The increase in the cell number in the S phase was less evident for 5-*O*-caffeoylquinic acid and 3,5-di-*O*-caffeoylquinic acid which, differently from the other compounds, were concomitant with a decrease in the percentage of Caco-2 cells in the G0/G1 phase.

In the SW480 cell line control (Figure 3b), the percentage of cells in G0/G1, S and G2/M phases was 69.2 ± 0.7%, 10.0 ± 0.5% and 13.8 ± 1.5%, respectively. Quercetin and quercetin-4′-*O*-glucoside promoted an evident increase in the number of SW480 cells in the S phase (increase of 3.1 and 2.5 times, respectively), concomitant to a significant decrease (*p <* 0.05) in the percentage of SW480 cells in the G0/G1 phase. A lower increase in the number of SW480 cells in the S phase was also recorded after treatments with the metabolites 3-(3′,4′-dihydroxyphenyl)acetic acid and 3-(3′-hydroxyphenyl)acetic acid.

### 2.4. Cell Metabolism of Phenolic Compounds and Metabolites

Most of the tested phenolic compounds underwent substantial biotransformations depending on their chemical structure and cell line.

In the SW480 cell line, quercetin-3-*O*-glucoside-4′-*O*-glucoside was extensively metabolized, its amount decreasing by 14.4%, 26.0% and 53.6% after 24, 48 and 72 h of incubation (Table 2).

Several new metabolites at *m/z* of 463.0888, 315.0513 and 301.0355 appeared already after 24 h of incubation. By comparing the *m/z* values, the fragmentation patterns and the retention times with authentic standards, the three compounds were identified as quercetin-3-*O*-glucoside, isorhamnetin (and the corresponding isomer 4′-*O*-methyl-quercetin) and quercetin, respectively. Similarly, quercetin-4′-*O*-glucoside was strongly metabolized to quercetin, isorhamnetin and 4′-*O*-methyl-quercetin, and quercetin metabolism resulted mainly in the formation of isorhamnetin and 4′-*O*-methyl-quercetin (Table 2). In these last two samples, a new compound having *m/z* 380.9921 was detected and identified as quercetin-*O*-sulphate. Concerning Caco-2 cells, quercetin-3-*O*-glucoside-4′-*O*-glucoside was found to be quite stable, without changes in its concentration during 72 h of incubation. Contrarywise, quercetin-4′-*O*-glucoside and quercetin cell medium concentrations had already strongly decreased during incubation with Caco-2 by 100% and 99.5%, respectively, after 24 h of incubation (Table 3). Nevertheless, no newly formed compounds were detected in the Caco-2 cell media.

As reported in Table 2 and Table 3, 3,5-di-*O*-caffeoylquinic acid, 3-*O*-caffeoylquinic acid and 5-*O*-caffeoylquinic acid underwent substantial isomerization during incubation with both the cell lines. Several positional isomers were already identified after 24 h of incubation of 3,5-di-*O*-caffeoylquinic acid with both the cell lines.

The two newly formed isomers detected in the highest amounts were 3,4-di-*O*-caffeoylquinic and 1,3-di-*O*-caffeoylquinic acids. Besides the isomerization, after incubation of 3,5-di-*O*-caffeoylquinic acid with both the cell lines, newly formed metabolites were detected; in particular, the different positional isomers of caffeoylquinic acids (i.e., 5-*O*-caffeoylquinic acid, 3-*O*-caffeoylquinic acid and 4-*O*-caffeoylquinic acid) deriving from the hydrolysis of a caffeoyl moiety from the di-*O*-caffeoylquinic acids. Similarly to 3,5-di-*O*-caffeoylquinic acid, 3-*O*-caffeoylquinic acid and 5-*O*-caffeoylquinic acid were also partially transformed in the corresponding positional isomers as well as suffering trans–cis isomerization (Table 2 and Table 3). After incubation with SW480, additional metabolites were detected such as caffeic acid (deriving from the hydrolysis of the quinic acid moiety from caffeoylquinic acids) and the different isomers of the methylated metabolites of feruloylquinic acids (Table 2). In addition, ferulic acid (deriving from the hydrolysis of the quinic acid moiety from feruloylquinic acids or from the methylation of caffeic acid) was detected after incubation of 5-*O*-caffeoylquinic acid with SW480 cells. No newly formed compounds were observed after incubation with Caco-2 cells.

Regarding the three tested colon metabolites, 3-(3′-hydroxyphenyl)acetic acid and 3-(3′-hydroxyphenyl)propanoic acid were quite stable after incubation with both the cell lines (Table 2 and Table 3). Contrariwise, a decrease in 3-(3′,4′-dihydroxyphenyl)acetic acid concentration was observed after incubation with both the cell lines, with the values plummeting to zero already after 24 h of incubation with Caco-2. No newly formed compounds were observed in SW480 cell medium, whereas a strong production of hydroxybenzoic acid was detected after incubation with Caco-2 (Table 3). Small amounts of hydroxybenzoic acid were also revealed after incubation of 3-(3′-hydroxyphenyl)propanoic acid with Caco-2 (Table 3).

## 3. Discussion

Flavonols (such as glycosylated quercetin derivatives) and hydroxycinnamic acids (such as chlorogenic acids) are among the most widespread phenolic compounds in foods and among the most important phenolic compounds introduced through the diet [18]. For example, onion, apple, tea and red wine are rich in glycosylated quercetin derivatives, whereas coffee, eggplant, artichoke, apple and cherry contain high amounts of chlorogenic acids [16,20,21,23,31,34,35].

A plethora of studies have suggested that dietary phenolic compounds, such as glycosylated quercetin derivatives and chlorogenic acids, are poorly absorbed in the stomach and small intestine [16,31,34,36]. For example, only 20–30% of hydroxycinnamic acids present in coffee or other foods such as yerba mate and artichoke are absorbed in the small intestine, and appeared in plasma mainly as phase II conjugated metabolites [16,34,37]. The absorption of quercetin derivatives in the small intestine is dependent on the chemical structure of the compound itself. For example, quercetin aglycone was poorly absorbed (about 20%) whereas quercetin mono-glucosides were better absorbed, reaching 50% of the ingested dose [31,36]. Therefore, unabsorbed phenolic compounds reach the colon, where the gut microbiota may metabolize the parent phenolic compounds, producing a broad assortment of low-molecular-weight phenolic acids, mainly hydroxyphenylacetic, hydroxyphenylpropanoic and hydroxybenzoic acids [38].

**Figure 4:**
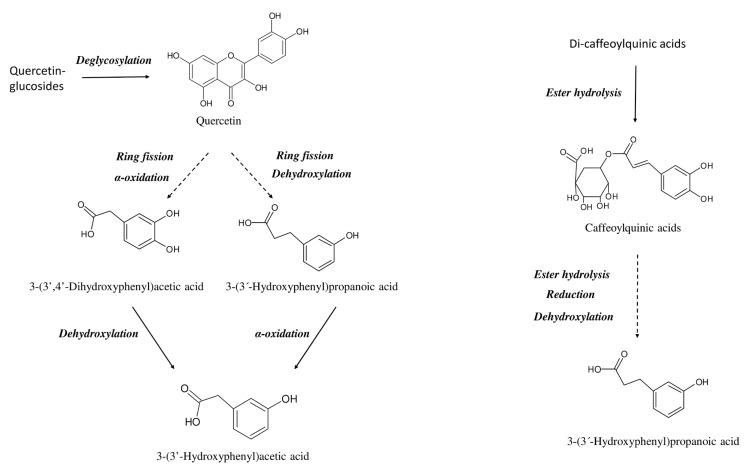
Simplified view of the colon metabolism of glycosylated quercetin derivatives and chlorogenic acids. Colon metabolism of glycosylated quercetin derivatives involves the hydrolysis of the glucose group, releasing the corresponding aglycone which undergoes C-ring fission followed by dehydroxylation or oxidation producing 3-(3′-hydroxyphenyl)propanoic acid and 3-(3′,4′-dihydroxyphenyl)acetic acid. The metabolism of these last compounds converges in the formation of 3-(3′-hydroxyphenyl)acetic acid. The colon metabolism of chlorogenic acids involved the hydrolysis of the quinic acid group, producing 3′,4′-dihydroxycinnamic acid, which is further reduced and dehydroxylated to 3-(3′-hydroxyphenyl)propanoic acid.

Colon metabolism of glycosylated quercetin derivatives begins with the hydrolysis of the glucoside moiety, releasing the corresponding aglycone that undergoes C-ring fission generating the transient intermediate 3-(3′,4′-dihydroxyphenyl)propanoic acid that is further dehydroxylated to 3-(3′-hydroxyphenyl)propanoic acid or oxidated to 3-(3′,4′-dihydroxyphenyl)acetic acid. These last compounds may finally be converted into 3-(3′-hydroxyphenyl)acetic acid by oxidation and dehydroxylation, respectively (Figure 4) [27,28,39]. Similarly, the colon metabolism of chlorogenic acids starts with the hydrolysis of the ester bond between quinic acid and hydroxycinnamic acid, producing 3′,4′-dihydroxycinnamic acid which is further reduced and dehydroxylated to 3-(3′-hydroxyphenyl)propanoic acid (Figure 4) [27,30]. Thus, the most important metabolites identified after colon fermentation of glycosylated quercetin derivatives are quercetin aglycone, 3-(3′,4′-dihydroxyphenyl)acetic acid, 3-(3′-hydroxyphenyl)acetic acid and 3-(3′-hydroxyphenyl)propanoic acid, whereas colon metabolism of chlorogenic acids mainly results in the accumulation of 3-(3′-hydroxyphenyl)propanoic acid [27,28,30,39,40].

A comparison of the data previously reported in the in vitro studies of colonic fermentation of quercetin and quercetin derivatives highlighted the great inter-individual variability in the gut metabolism of these compounds.

For example, Juániz et al. [40] and Cattivelli et al. [28] identified 3-(3′-hydroxyphenyl)propanoic acid as the main metabolite produced after fecal fermentation of glycosylated quercetins in green pepper and red-skinned onion. On the other hand, Di Pede et al. [39] pointed out 3-(3′,4′-dihydroxyphenyl)acetic acid as the metabolite generated in higher amounts after quercetin fermentation by gut microbiota. Contrary to this, Serra et al. [27] detected 3-(3′-hydroxyphenyl)acetic acid as the major colon metabolite after fermentation of quercetin and quercetin-rhamnoside, and found almost equimolar amounts of 3-(3′-hydroxyphenyl)acetic acid and 3-(3′,4′-dihydroxyphenyl)acetic acid after fermentation of quercetin-rutinoside. In another study, Jaganath et al. [41] compared the colon metabolism of quercetin-rutinoside among three individual donors. Only one donor was able to produce both 3-(3′-hydroxyphenyl)acetic acid and 3-(3′,4′-dihydroxyphenyl)acetic acid, whereas the other two donors generated only 3-(3′,4′-dihydroxyphenyl)acetic acid. There were also differences in the rate of quercetin metabolic conversion. In some studies, quercetin aglycone was still present in high concentrations after 5-6 h of fermentation whereas, in others, quercetin completely disappeared after a few hours of fecal fermentation [28,39,40,41,42]. Therefore, inter-individual variability determined not only the type and concentration of produced metabolites but also the rate of bioconversion of quercetin. Differently, chlorogenic acid metabolism seemed to be less sensitive to inter-individual variability, with 3-(3′-hydroxyphenyl)propanoic acid being the main metabolite identified in the majority of the studies [29,30,43]. However, there were also differences in the metabolic rate in this case. In some studies, chlorogenic acids totally disappeared after a few hours of incubation with colon bacteria, whereas in other studies they were found still present after 10 or 24 h of incubation [29,30,43,44,45]. Therefore, it can be inferred that both parent phenolic compounds and their colon metabolites co-exist in the colon, especially after repeated intake of phenolic-rich foods. Moreover, the relative amount of parent phenolic compounds and produced metabolites is greatly influenced by the individual composition of gut microbiota, showing great inter-individual variability [30,33].

In this study, we found that both parent compounds (i.e., glycosylated quercetin derivatives, quercetin aglycone and chlorogenic acids) as well as their most important colon metabolites were able to inhibit colon cancer cell proliferation at a concentration of 100 μmol/L. The tested concentration of 100 μmol/L is relevant from a physiological point-of-view, especially for the colon metabolites.

Previous studies reported in human fecal water or human fecal matrix a broad range of concentrations for the tested colon metabolites according to the inter-individual variability. Karlsson et al. [46] reported a concentration of 61 ± 34 μmol/L in human fecal water, whereas Jenner et al. [47] stated a mean value of 68 ± 93 μmol/L (range 0.72–209 μmol/L) for the colon metabolite 3-(3′-hydroxyphenyl)propanoic acid. Higher values of 3-(3′-hydroxyphenyl)propanoic acid (between 15 to 1076 μmol/L) were described by Knust et al. [48] in human fecal matrix. Lower concentrations were found for the colon metabolite 3-(3′-hydroxyphenyl)acetic acid (mean values from 30 to 110 μmol/L), with a wide range among individuals (from 4 to 294 μmol/L) and for 3-(3′,4′-dihydroxyphenyl)acetic acid (mean values from 7 to 64 μmol/L) and an individual range from 1 to 277 μmol/L [47,48,49]. It is important to note that the discussed studies have been carried out without any dietary restriction or supplementation, and thus represent values commonly found in a normal dietary regimen. Supplementation of phenolic-rich foods such as raspberry may further increase the amount of phenolic metabolites, as observed by Gill et al. [49] who found in human fecal water maximum values of 1810, 442 and 327 μmol/L of 3-(3′-hydroxyphenyl)propanoic acid, 3-(3′,4′-dihydroxyphenyl)acetic acid and 3-(3′-hydroxyphenyl)acetic acid. No available data were present in the literature about the fecal concentration of quercetin and its derivatives as well as of chlorogenic acids. However, as reported above, in vitro studies have suggested their presence in the colon in co-existence with their colon metabolites, especially following repeated ingestion of quercetin-rich or chlorogenic-acid-rich foods.

The anti-proliferative effect was time-dependent and increased in conjunction with increasing incubation time. The parent compounds 3,5-di-*O*-caffeoylquinic acid, 3-*O*-caffeoylquinic acid and 5-*O*-caffeoylquinic acid, as well as the metabolite 3-(3′,4′-dihydroxyphenyl)acetic acid, were the most active against Caco-2 cell proliferation at any incubation time. On the contrary, the parent compounds quercetin, quercetin-4′-*O*-glucoside and quercetin-3-*O*-glucoside-4′-*O*-glucoside, as well as the metabolites 3-(3′,4′-dihydroxyphenyl)acetic acid and 3-(3′-hydroxyphenyl)acetic acid, were the most active against SW480 cell proliferation.

In Caco-2 cells, the extent of the anti-proliferative effect seemed to be related to the presence of specific structural motifs. The most active molecules, i.e., 3,5-di-*O*-caffeoylquinic acid, 3-*O*-caffeoylquinic acid, 5-*O*-caffeoylquinic acid, 3-(3′,4′-dihydroxyphenyl)acetic acid and quercetin, displayed in their structure a catechol-type moiety, which was fundamental for exerting an anti-proliferative effect. For example, 3-(3′,4′-dihydroxyphenyl)acetic acid bearing a catechol moiety was clearly more active than the mono-hydroxylate 3-(3′-hydroxyphenyl)acetic acid. Similarly, quercetin showed a higher anti-proliferative activity than the glycosylated derivatives quercetin-4′-*O*-glucoside and quercetin-3-*O*-glucoside-4′-*O*-glucoside, where one of the two hydroxyl groups of the catechol moiety is esterified with a glucose molecule. Also, the presence of one or more glucose residues hampered the anti-proliferative effect, since quercetin showed a significantly higher effect than quercetin-4′-*O*-glucoside whereas quercetin-3-*O*-glucoside-4′-*O*-glucoside was not active at any time. The effect of the tested compounds was quite different when incubated with SW480. In this case, quercetin and its glycosylated derivatives together with the metabolites 3-(3′,4′-dihydroxyphenyl)acetic acid and 3-(3′-hydroxyphenyl)acetic acid were the most active compounds, whereas the tested chlorogenic acids, although they present a certain degree of inhibition, were less effective in inhibiting SW480 proliferation.

Several previous studies reported the anti-proliferative effect of chlorogenic acids against human colon cancer cells. For example, 3-*O*-caffeoylquinic acid was found to be able to reduce Caco-2 and HT29 colon cancer cell proliferation, whereas dicaffeoylquinic acids inhibited the growth of HT29 and DLD-1 cancer cells [50,51,52].

Moreover, 3-(3′,4′-dihydroxyphenyl)acetic acid was found to be active against the proliferation of human colon cancer cells HCT116 and SNUC4 [53,54]. Furthermore, quercetin has already been found to be active against Caco-2 proliferation (as well as in other colon cancer cell models) at concentrations similar to those tested in this study [55,56].

As already mentioned, parent phenolic compounds and metabolites may co-exist in the colon and the ratio of parent phenolic compounds/metabolites is strongly dependent on the inter-individual variability related to the ability to metabolize the parent phenolic compounds slowly or rapidly. To better understand the potential impact of inter-individual variability, different mixtures of parent compounds and colon metabolites were prepared to mimic the low-producers and high-producers metabotypes. The quercetin derivative/metabolite mixes behaved differently depending on the tested cell line. The cell growth inhibition increased passing from the mix with the low amount of metabolites (i.e., low-producers) to the mix with the highest amount of metabolites (i.e., high-producers) after incubation with the Caco-2 cell line, whereas the trend was opposite for the SW480 cell line. However, the high-producers mix was still able to inhibit SW480 cell proliferation with values near to 50% at 200 μmol/L concentration. For the chlorogenic acid/metabolite mixes, the effect was more pronounced for the low-producers mix with respect to the high-producers mix in Caco-2 cells; nevertheless, the inhibitory effect of the high-producers mix was near to 50% at 200 μmol/L concentration. No difference in the anti-proliferative activity was observed in the SW480 cell line with the three chlorogenic acid/metabolite mixes. Therefore, our data support the hypothesis that parent phenolic compounds (i.e., quercetin derivatives and chlorogenic acids), once present in the colon after the consumption of phenolic-rich vegetables, may exert anti-proliferative activity against colon cancer cells. Later, when they begin to be metabolized, the formed colon metabolites may sustain and prolong the inhibitory effect towards colon cancer cell proliferation.

Cell cycle analysis pointed out that all the tested compounds arrest the cell cycle at the S phase in both the cell lines. The significant increase in the Caco-2 cell number in the S phase was concomitant with a decrease in the amount of cells in G2/M phase, indicating that quercetin, 3-*O*-caffeoylquinic acid and 3-(3′,4′-dihydroxyphenyl)acetic acid inhibited cell proliferation by arresting the cell cycle at the S phase, thus preventing Caco-2 cells from entering the G2 phase. In the case of SW480, the increase in the percentage of cells in the S phase was associated with a reduction in cell number for the G0/G1 phase.

Previous studies have found that quercetin and chlorogenic acid induced cell cycle arrest at the S phase in several colon cancer cell lines, including Caco-2 and SW480 [52,57]. Differently from this study, 3-(3′,4′-dihydroxyphenyl)acetic acid promoted cell cycle arrest at the G0/G1 phase in HT-29 colon cancer cells [58].

The different tested phenolic compounds and metabolites displayed different stability in the cell media and were differently metabolized accordingly to their structure and the specific cell line. Quercetin and glycosylated quercetin derivatives were strongly metabolized by SW480 cells, whereas no newly formed metabolites were detected after incubation with Caco-2 cells. In SW480 cells, quercetin-3-*O*-glucoside-4′-*O*-glucoside underwent extensive deglycosylation, suggesting the presence in these cells of a membrane-bound β-glucosidase. Moreover, the appearance of only the 3-*O*-glucoside derivative of quercetin suggested a strong preference of this β-glucosidase for the glycosidic linkage at the 4′-*O* position rather than the 3-*O* position. This specificity was confirmed also by the data about quercetin-4′-*O*-glucoside metabolism in SW480 cells. This compound was rapidly hydrolyzed after 24 h in an equimolar amount of the corresponding aglycone quercetin. Furthermore, the fastest deglycosylation of quercetin-4′-*O*-glucoside with respect to quercetin-3-*O*-glucoside-4′-*O*-glucoside indicated that the presence of a 3-*O*-glucoside moiety in the molecule hampered the hydrolyzing ability of SW480 β-glucosidase. The appearance of small amounts of quercetin after 48 h of incubation of quercetin-3-*O*-glucoside-4′-*O*-glucoside pointed out the presence, also, of a β-glucosidase with specificity towards the 3-*O*-glucosidic linkage or the ability of the same β-glucosidase to also hydrolyze with less efficacy the 3-*O*-glucosidic linkage. Additional metabolites—and, in particular, isorhamnetin, 4′-*O*-methyl quercetin and quercetin-*O*-sulphate—were detected after incubation of quercetin and its derivatives with SW480 cell lines, suggesting the presence in these cells of a catechol-*O*-methyltransferase and a sulfotransferase. The appearance of higher amounts of isorhamnetin with respect to 4′-*O*-methyl quercetin pointed out a preference for the OH in position C3 for the catechol-*O*-methyltransferase. Due to the lack of authentic standards, it was not possible to identify the specific isomer of quercetin-sulphate. On the contrary, no β-glucosidase, as well as catechol-*O*-methyltransferase and sulfotransferase, activities were observed in Caco-2 cells after incubation with quercetin and glycosylated quercetin derivatives. In this cell line, quercetin-3-*O*-glucoside-4′-*O*-glucoside was stable in the cell media with a 100% recovery also after 72 h of incubation, whereas quercetin and quercetin-4′-*O*-glucoside rapidly disappeared already after 24 h of incubation. Since neither newly formed metabolites nor the known quercetin oxidation products were detected in the cell media, the most plausible explanation was that quercetin and quercetin-4′-*O*-glucoside were rapidly absorbed by Caco-2 cells. Previous studies demonstrated active absorption of quercetin and quercetin-4′-*O*-glucoside by Caco-2 cells [59]. The same consideration can be made for SW480 cells, where the final recovery of quercetin and its metabolites after 72 h of incubation of quercetin and quercetin-4′-*O*-glucoside was below 100%.

The cell metabolism of glycosylated quercetin derivatives may have an impact on their anti-proliferative activity. In SW480, quercetin was the most active compound and can also be considered responsible for the anti-proliferative activity of quercetin-4′-*O*-glucoside, since this last compound is rapidly hydrolyzed to the corresponding aglycone. Similarly, quercetin-3-*O*-glucoside-4′-*O*-glucoside was hydrolyzed to quercetin-3-*O*-glucoside, which can be responsible for its anti-proliferative effect against SW480 cells. The anti-proliferative activity of quercetin-3-*O*-glucoside has been already reported by several authors [60].

Likewise to what was noted for quercetins, chlorogenic acids were also extensively metabolized by SW480, whereas no metabolites were found after incubation with Caco-2. In addition to the obvious isomerization (acyl migration), which is dependent on the pH and not on the presence of cells [61], both 3,5-di-*O*-caffeoylquinic acid and the two caffeoylquinic acids (and their isomers) were hydrolyzed to caffeoylquinic acids and caffeic acid, respectively, suggesting the presence of esterase activity in SW480 cells [62]. Moreover, caffeoylquinic acids were methylated by SW480 catechol-*O*-methyltransferase, producing different isomers of feruloylquinic acids. In the Caco-2 cell line, 3,5-di-*O*-caffeoylquinic acid, 3-*O*-caffeoylquinic acid and 5-*O*-caffeoylquinic acid almost totally disappeared from the cell media already after 24 h of incubation. Some studies suggest that caffeoylquinic and dicaffeoylquinic acids can be transported in Caco-2 cells [61,63].

Concerning the phenolic metabolites, both 3-(3′-hydroxyphenyl)acetic acid and 3-(3′-hydroxyphenyl)propanoic acid were stable after incubation with Caco-2 and SW480, with recoveries of more than 80% after 72 h of incubation. On the contrary, 3-(3′,4′-dihydroxyphenyl)acetic acid concentration dropped to zero already after 24 h of incubation with Caco-2 cells. The only identified metabolite was hydroxybenzoic acid, which accounted for about the 30% of the initial amount of 3-(3′,4′-dihydroxyphenyl)acetic acid after 72 h of incubation. It is possible that this metabolite was formed by two consecutive reactions of de-hydroxylation and α-oxidation (or vice versa), which implies the uptake of 3-(3′,4′-dihydroxyphenyl)acetic in Caco-2 cells, as already suggested [64].

## 4. Materials and Methods

### 4.1. Materials

Chemicals and materials for cell culture were purchased from VWR International (Milan, Italy). The MTS cell proliferation assay kit was obtained from Promega (Milan, Italy). Mass spectrometry solvents were purchased from Bio-Rad (Hercules, CA, USA). The parent phenolic compounds 3-*O*-caffeoylquinic acid (purity ≥ 99%), 5-*O*-caffeoylquinic acid (purity ≥ 99%), 3,5-*O*-dicaffeoylquinic acid (purity ≥ 97%), quercetin (purity ≥ 99%), quercetin-4′-*O*-glucoside (purity ≥ 99%) and quercetin-3-*O*-glucoside-4′-*O*-glucoside (purity ≥ 98.5%) were obtained from Extrasynthese (Genay, France). Phenolic compound metabolites 3-(3′-hydroxyphenyl)propanoic acid (purity ≥ 98%), 3-(3′-hydroxyphenyl)acetic acid (purity ≥ 99%) and 3-(3′,4′-dihydroxyphenyl)acetic acid (purity ≥ 98%) were purchased from Thermo-Fisher Scientific (Waltham, MA, USA). All the other chemicals were purchased from Sigma-Aldrich (Milan, Italy).

### 4.2. Cell Cultures, Phenolic Compound Preparation and Anti-Proliferative Assay

Human colon cancer Caco-2 and SW480 cell lines were cultured and grown as previously reported [62]. All the phenolic compounds were dissolved in dimethyl-sulfoxide (DMSO) at a concentration of 50 mmol/L, diluted in the respective cell culture media at a concentration of 100 μmol/L (final DMSO concentration of 0.5%) and finally filtered at 0.2 μm.

For the anti-proliferative assay, cells were seeded in 96-well plates in the amount of 4000 and 8000 cells/cm^2^ for Caco-2 and SW480, respectively. Cells were then left to adhere for 24 h before the addition of 200 μL of phenolic compounds (100 μmol/L). Incubation was carried out for 24, 48 and 72 h. Data were compared with a control solution containing 0.5% DMSO in cell medium and representing 100% proliferation. In addition, six different mixes were prepared to simulate the simultaneous presence of parent phenolic compounds and metabolites by mimicking the possible human inter-individual variability in colon metabolism of phenolic compounds. Two sets of mixes were prepared to consider the colon metabolism of quercetins and chlorogenic acids. The quercetin mixtures (QUE mixes) contained different amounts of quercetin-3-*O*-glucoside-4′-*O*-glucoside, quercetin-4′-*O*-glucoside and quercetin (the parent compounds) as well as the respective metabolites (3-(3′-hydroxyphenyl)acetic acid, 3-(3′,4′-dihydroxyphenyl)acetic acid and 3-(3′-hydroxyphenyl)propanoic acid). The chlorogenic acids mixtures (CGA mixes) were instead prepared by mixing the respective parent compounds (3-*O*-caffeoylquinic acid, 5-*O*-caffeoylquinic acid and 3,5-*O*-dicaffeoylquinic acid) with the metabolite 3-(3′-hydroxyphenyl)propanoic acid. For each set of mixtures (QUE and CGA mixtures), three different mixes were formulated. One set of mixes mimicked the low-producers metabotype and contained 90% parent compounds and 10% metabolites (mixes QUE-LP and CGA-LP). Another set of mixes was prepared to simulate the high-producers metabotype and contained 10% parent compounds and 90% metabolites (mixes QUE-HP and CGA-HP). Finally, a third set of mixes was prepared with equimolar amounts of all the compounds (mixes QUE-EQ and CGA-EQ). The anti-proliferative activity of the mixes was determined by using the final concentrations of 100 and 200 μmol/L (sum of the concentrations of the compounds present in the mixes). The composition of each mix is reported in Table 4.

Cell proliferation was assessed by the MTS assay as previously described [63].

For the most active compounds, the IC_50_ values (defined as the phenolic compound concentration able to inhibit cell proliferation by 50%) were calculated by carrying out the anti-proliferative activity assay with different concentrations of the specific phenolic compounds (ranging from 1.5 to 200 μmol/L). The IC_50_ values were calculated through non-linear regression analysis by plotting the base-10 logarithm of the phenolic compound concentration vs. the percentage of inhibition.

### 4.3. Cell Cycle Analysis

Cell cycle analysis was carried out in both Caco-2 and SW480 cell lines as previously reported by Nicoletti et al. [65]. Cells were seeded in 6-well plates at a density of 4000 and 8000 cells/cm^2^ for Caco-2 and SW480, respectively. Cells were then left to adhere for 24 h before the addition of 200 μL of the selected phenolic compounds at a concentration equal to the calculated IC_50_ values at 72 h of incubation. The tested phenolic compounds were 3-*O*-caffeoylquinic acid, 5-*O*-caffeoylquinic acid, 3,5-*O*-dicaffeoylquinic acid, quercetin and 3-(3′,4′-dihydroxyphenyl)acetic acid for Caco-2, and quercetin, quercetin-4′-*O*-glucoside, 3-(3′-hydroxyphenyl)acetic acid and 3-(3′,4′-dihydroxyphenyl)acetic acid for SW480.

After 72 h of incubation, the cells were washed with PBS, detached from the 6-well plates with 500 μL of trypsin and resuspended in 1000 μL of medium. Subsequently, they were centrifuged at 3500 rpm for 5 min. After removing the supernatant, the pellet was resuspended with 400 μL of Nicoletti’s solution (sodium citrate 0.1%, Triton X-100 0.1% and 20 μg/mL propidium iodide). After 15 min of incubation at 4 °C in the dark, cell cycle analysis was performed with an Epics XL MCL flow cytometer (Beckman Coulter, Brea, CA, USA).

The data were compared with a control solution containing 0.5% DMSO in the cell medium.

### 4.4. High-Resolution Mass Spectrometry Analysis of Cell Media

To analyze the metabolism of phenolic compounds by Caco-2 and SW480 cells, the cell culture media were withdrawn after 0, 24, 48 and 72 h of incubation. Phenolic compounds were extracted from the culture media by following the procedure reported in Sala et al. [66]. Briefly, 100 μL of cold methanol was mixed with 100 μL of cell media and vortexed for 1 min. The mixture was then centrifuged at 10,000× *g* (4 °C, 5 min) and the supernatant was injected, after appropriate dilution, in an ultra-high-performance liquid chromatography high-resolution mass spectrometry system (UHPLC-HR-MS).

The HR-MS system consisted of a UHPLC Ultimate 3000 separation module, equipped with a C18 column (Acquity UPLC HSS C18 reversed phase, 2.1 × 100 mm, 1.8 μm particle size, Waters, Milan, Italy) and coupled with a Q Exactive Hybrid Quadrupole-Orbitrap Mass Spectrometer (Thermo Scientific, San Jose, CA, USA). Mobile phases consisted of a mixture of water containing formic acid (0.1%) (mobile phase A) and acetonitrile containing formic acid (0.1%) (mobile phase B). The gradient began at 1% B, reaching 40% B over 20 min. To wash the column, the mobile phase B concentration was raised to 99% over 6 min and kept at 99% for 3 min before returning to the initial conditions. The MS and MS/MS conditions are fully described in Martini et al. [67].

Quantification was carried out by building external calibration curves with the available standard compounds.

### 4.5. Statistical Analysis

All data are reported as mean ± standard deviation (SD) for three replicates for each prepared compound. Univariate analysis of variance (ANOVA) with Tukey post hoc test was applied using GraphPad Prism 6.0 (GraphPad Software, San Diego, CA, USA). The differences were considered significant with *p* < 0.05. The IC_50_ values were calculated by using GraphPad Prism 6.0.

## 5. Conclusions

For the first time, the anti-proliferative activity of the dietarily most relevant chlorogenic acids, quercetin derivatives, and their colon microbial metabolites against two different human colon cancer cell models was tested and compared. Generally, the reported data demonstrate that in-vivo-pertinent mixtures of parent compounds and metabolites at concentrations attainable in the human colon following the consumption of phenolic-rich vegetables exert anti-proliferative effects against colon cancer adenocarcinoma cell lines. Since the anti-proliferative activity was nearly comparable between mixes that mirror the low-producers and high-producers human metabotypes, it can be speculated that inter-individual variability does not substantially affect the potential in vivo anti-cancer activity of phenolic compounds.

The main limitation of this study is related to the use of 2D cell models that, somewhat, do not reflect the physiological tumor microenvironment with respect to more complex in vitro models such as 3D tumor spheroid models. Future research should be carried out to provide an even greater physiological significance to the reported data by using 3D colon cancer cell models.

However, these results suggest that gut microbiota metabolites may sustain the anti-proliferative effects observed for the parent compounds, playing an important role in the protective effect of phenolic compounds against colon cancer. The present manuscript, therefore, contributes to unveiling the fundamental role of the colonic metabolism in the context of the protective effect of phenolic compounds against colon cancer.

## Figures and Tables

**Table 1:** IC_50_ values (μmol/L) of the different tested compounds after 24, 48 and 72 h of incubation with Caco-2 and SW480 cell lines.

Compound	Caco-2 (μmol/L)			SW 480 (μmol/L)		
	24 h	48 h	72 h	24 h	48 h	72 h
Quercetin-3-*O*-glucoside-4′-*O*-glucoside	n.a.	n.a.	n.a.	n.a.	>200	~200
Quercetin-4′-*O*-glucoside	n.a.	>200	>200	n.a.	144.3 ± 6.6	102.2 ± 5.3 ^#^
Quercetin	n.a.	>200	116.1 ± 4.9	n.a.	89.2 ± 3.1	58.2 ± 2.7
3-*O*-Caffeoylquinic acid	97.3 ± 2.9 ^#^	42.8 ± 1.7 *	40.4 ± 2.0 *	n.a.	>200	>200
5-*O*-Caffeoylquinic acid	169.5 ± 8.3	98.8 ± 3.0 ^#^	31.3 ± 2.1	n.a.	>200	>200
3,5-di-*O*-Caffeoylquinic acid	>200	27.5 ± 1.0	8.8 ± 0.2	n.a.	>200	>200
3-(3′-Hydroxyphenyl)acetic acid	n.a.	> 200	> 200	n.a.	>200	99.6 ± 4.8 ^#^
3-(3′,4′-Dihydroxyphenyl)acetic acid	79.5 ± 1.4	14.7 ± 0.8	3.0 ± 0.1	n.a.	>200	92.2 ± 6.9 ^#^
3-(3′-Hydroxyphenyl)propanoic acid	>200	>200	>200	n.a.	n.a.	>200

n.a. means not active compound. Number with the same superscript symbol (* or #) were not significantly different (*p* > 0.05). The maximum tested concentration was 200 μmol/L.

**Table 2:** Changes in the concentration of tested phenolic compounds and in the newly formed metabolites after 24, 48 and 72 h of incubation with the SW480 cell line.

Compound		SW 480 (μmol/L)			
		0 h	24 h	48 h	72 h
Substrate	Quercetin-3-*O*-glucoside-4′-*O*-glucoside	50	42.77 ± 0.10	37.04 ± 0.35	23.22 ± 0.22
Metabolite	Quercetin-3-*O*-glucoside		3.35 ± 0.01	8.94 ± 0.03	9.34 ± 0.05
	Quercetin		n.d.	0.04 ± 0.00	0.07 ± 0.00
	Isorhamnetin		n.d.	0.01 ± 0.00	0.01 ± 0.00
	4′-*O*-methylquercetin		n.d.	0.01 ± 0.00	0.01 ± 0.00
					
Substrate	Quercetin-4′-*O*-glucoside	50	2.98 ± 0.07	0.07 ± 0.00	0.01 ± 0.00
Metabolite	Quercetin		52.70 ± 2.24	36.89 ± 0.52	12.78 ± 0.41
	Isorhamnetin		n.d.	0.79 ± 0.03	1.03 ± 0.04
	4′-*O*-methylquercetin		n.d.	0.49 ± 0.01	0.71 ± 0.04
	Quercetin-*O*-sulphate		n.d.	0.06 ± 0.00	0.12 ± 0.01
					
					
Substrate	Quercetin	50	47.39 ± 1.22	40.37 ± 1.20	14.83 ± 0.84
Metabolite	Isorhamnetin		1.61 ± 0.01	3.46 ± 0.08	3.76 ± 0.11
	4′-*O*-methylquercetin		0.97 ± 0.04	1.65 ± 0.04	1.83 ± 0.25
	Quercetin-*O*-sulphate		0.26 ± 0.01	0.48 ± 0.01	0.61 ± 0.01
					
Substrate	3-*O*-Caffeoylquinic acid *trans*	50	23.34 ± 0.07	19.54 ± 0.07	19.83 ± 0.05
Metabolite	5-*O*-Caffeoylquinic acid *trans*		4.95 ± 0.04	7.37 ± 0.07	11.32 ± 0.43
	4-*O*-Caffeoylquinic acid *trans*		6.89 ± 0.06	8.07 ± 0.24	12.02 ± 0.24
	3-*O*-Caffeoylquinic acid *cis*		4.72 ± 0.04	2.51 ± 0.01	3.27 ± 0.17
	Caffeic acid		0.44 ± 0.01	0.43 ± 0.01	0.57 ± 0.01
	3-*O*-Feruloylquinic acid		0.08 ± 0.00	0.12 ± 0.00	0.17 ± 0.00
	4-*O*-Feruloylquinic acid		0.02 ± 0.00	0.04 ± 0.00	0.07 ± 0.00
	5-*O*-Feruloylquinic acid		0.03 ± 0.00	0.03 ± 0.00	0.05 ± 0.00
					
Substrate	5-*O*-Caffeoylquinic acid *trans*	50	16.76 ± 0.01	17.52 ± 0.08	13.96 ± 0.03
Metabolite	5-*O*-Caffeoylquinic acid *cis*		2.50 ± 0.01	0.97 ± 0.05	1.42 ± 0.01
	3-*O*-Caffeoylquinic acid *trans*		2.21 ± 0.05	5.14 ± 0.07	5.55 ± 0.09
	4-*O*-Caffeoylquinic acid *trans*		12.69 ± 0.12	14.80 ± 0.09	11.93 ± 0.03
	3-*O*-Caffeoylquinic acid *cis*		0.41 ± 0.05	0.36 ± 0.04	0.72 ± 0.03
	Caffeic acid		n.d.	0.08 ± 0.00	0.09 ± 0.01
	3-*O*-Feruloylquinic acid		0.06 ± 0.00	0.07 ± 0.00	0.05 ± 0.00
	4-*O*-Feruloylquinic acid		0.04 ± 0.00	0.05 ± 0.00	0.04 ± 0.00
	5-*O*-Feruloylquinic acid		0.01 ± 0.00	0.03 ± 0.00	0.03 ± 0.00
	Ferulic acid		0.06 ± 0.02	0.33 ± 0.01	0.37 ± 0.01
					
Substrate	3,5-di-*O*-Caffeoylquinic acid	50	7.13 ± 0.13	5.41 ± 0.07	6.17 ± 0.05
Metabolite	1,3-di-*O*-Caffeoylquinic acid		8.97 ± 0.04	8.87 ± 0.05	10.98 ± 0.02
	3,4-di-*O*-Caffeoylquinic acid		10.12 ± 0.10	8.04 ± 0.14	9.05 ± 0.15
	4,5-di-*O*-Caffeoylquinic acid		3.30 ± 0.05	2.20 ± 0.02	1.71 ± 0.03
	1,4-di-*O*-Caffeoylquinic acid		0.91 ± 0.03	0.66 ± 0.03	0.55 ± 0.01
	3-*O*-Caffeoylquinic acid *trans*		0.09 ± 0.01	0.13 ± 0.01	0.27 ± 0.01
	4-*O*-Caffeoylquinic acid *trans*		0.07 ± 0.01	0.12 ± 0.01	0.25 ± 0.01
	5-*O*-Caffeoylquinic acid *trans*		0.08 ± 0.01	0.14 ± 0.01	0.29 ± 0.01
	Caffeic acid		n.d.	n.d.	1.07 ± 0.01
					
Substrate	3-(3′-hydroxyphenyl)acetic acid	50	42.23 ± 0.55	42.81 ± 0.10	39.28 ± 0.85
					
Substrate	3-(3′,4′-dihydroxyphenyl)acetic acid	50	21.62 ± 0.48	15.84 ± 0.09	10.30 ± 0.36
					
Substrate	3-(3′-hydroxyphenyl)propanoic acid	50	52.74 ± 0.44	50.70 ± 0.11	49.95 ± 0.37

n.d. means not detected.

**Table 3:** Changes in the concentration of tested phenolic compounds and in the newly formed metabolites after 24, 48 and 72 h of incubation with the Caco-2 cell line.

Compound		Caco-2 (μmol/L)			
		0 h	24 h	48 h	72 h
Substrate	Quercetin-3-*O*-glucoside-4′-*O*-glucoside	50	53.05 ± 0.44	50.05 ± 0.32	50.18 ± 0.66
					
Substrate	Quercetin-4′-*O*-glucoside	50	n.d.	n.d.	n.d.
					
					
Substrate	Quercetin	50	0.61 ± 0.01	0.29 ± 0.01	0.52 ± 0.02
					
Substrate	3-*O*-Caffeoylquinic acid *trans*	50	2.80 ± 0.16	0.04 ± 0.00	0.02 ± 0.00
Metabolite	5-*O*-Caffeoylquinic acid *trans*		2.71 ± 0.09	0.02 ± 0.00	0.01 ± 0.00
	4-*O*-Caffeoylquinic acid *trans*		2.93 ± 0.10	0.03 ± 0.00	0.02 ± 0.00
	3-*O*-Caffeoylquinic acid *cis*		0.26 ± 0.01	0.02 ± 0.00	0.02 ± 0.00
					
Substrate	5-*O*-Caffeoylquinic acid *trans*	50	2.70 ± 0.01	0.02 ± 0.00	0.02 ± 0.00
Metabolite	5-*O*-Caffeoylquinic acid *cis*		0.12 ± 0.01	0.01 ± 0.00	0.01 ± 0.00
	3-*O*-Caffeoylquinic acid *trans*		2.35 ± 0.02	0.21 ± 0.00	0.19 ± 0.00
	4-*O*-Caffeoylquinic acid *trans*		3.46 ± 0.03	0.09 ± 0.00	0.09 ± 0.00
	3-*O*-Caffeoylquinic acid *cis*		0.15 ± 0.01	0.02 ± 0.00	0.02 ± 0.00
					
Substrate	3,5-di-*O*-Caffeoylquinic acid	50	0.15 ± 0.00	0.08 ± 0.00	0.18 ± 0.00
Metabolite	1,3-di-*O*-Caffeoylquinic acid		0.33 ± 0.01	0.16 ± 0.00	0.39 ± 0.00
	3,4-di-*O*-Caffeoylquinic acid		0.32 ± 0.00	0.18 ± 0.00	0.36 ± 0.00
	4,5-di-*O*-Caffeoylquinic acid		0.06 ± 0.00	0.03 ± 0.00	0.03 ± 0.00
	1,4-di-*O*-Caffeoylquinic acid		n.d.	n.d.	0.10 ± 0.00
					
Substrate	3-(3′-hydroxyphenyl)acetic acid	50	48.83 ± 0.85	47.02 ± 0.39	43.17 ± 0.06
					
Substrate	3-(3′,4′-dihydroxyphenyl)acetic acid	50	n.d.	n.d.	n.d.
	Hydroxybenzoic acid		n.d.	3.51 ± 0.07	15.73 ± 0.37
					
Substrate	3-(3′-hydroxyphenyl)propanoic acid	50	45.51 ± 0.41	46.27 ± 0.58	46.28 ± 0.76
	Hydroxybenzoic acid		n.d.	0.31 ± 0.01	0.30 ± 0.01

n.d. means not detected.

**Table 4:** Composition of the different mixes tested for their anti-proliferative effect against human colon carcinoma cell lines.

Compound	QUE-LP	QUE-EQ	QUE-HP	CGA-LP	CGA-EQ	CGA-HP
						
Quercetin-3-*O*-glucoside-4′-*O*-glucoside	60 μmol/L	33.3 μmol/L	6.7 μmol/L	/	/	/
Quercetin-4′-*O*-glucoside	60 μmol/L	33.3 μmol/L	6.7 μmol/L	/	/	/
Quercetin	60 μmol/L	33.3 μmol/L	6.7 μmol/L	/	/	/
3-*O*-Caffeoylquinic acid	/	/	/	60 μmol/L	50 μmol/L	6.7 μmol/L
5-*O*-Caffeoylquinic acid	/	/	/	60 μmol/L	50 μmol/L	6.7 μmol/L
3,5-di-*O*-Caffeoylquinic acid	/	/	/	60 μmol/L	50 μmol/L	6.7 μmol/L
3-(3′-Hydroxyphenyl)acetic acid	6.7 μmol/L	33.3 μmol/L	60 μmol/L	/	/	/
3-(3′,4′-Dihydroxyphenyl)acetic acid	6.7 μmol/L	33.3 μmol/L	60 μmol/L	/	/	/
3-(3′-Hydroxyphenyl)propanoic acid	6.7 μmol/L	33.3 μmol/L	60 μmol/L	20 μmol/L	50 μmol/L	180 μmol/L

The composition refers to the mixes prepared at the final concentration of 200 μmol/L considering all the added compounds. In the mixes prepared at the final concentration of 100 μmol/L, the concentration of each compound was halved.

## Data Availability

The data presented in this study are available on request from the corresponding author.
